# Ten simple rules for supporting historically underrepresented students in science

**DOI:** 10.1371/journal.pcbi.1009313

**Published:** 2021-09-16

**Authors:** Suchinta Arif, Melanie Duc Bo Massey, Natalie Klinard, Julie Charbonneau, Loay Jabre, Ana Barbosa Martins, Danielle Gaitor, Rhiannon Kirton, Catalina Albury, Karma Nanglu

**Affiliations:** 1 Department of Biology, Dalhousie University, Halifax, Canada; 2 Earth to Ocean Research Group, Simon Fraser University, Burnaby, Canada; 3 Department of Medical Neurosciences, Dalhousie University, Halifax, Canada; 4 Department of Geography, Western University, London, Canada; 5 Department of Paleobiology, Smithsonian National Museum of Natural History, Washington, District of Columbia, United States of America; Carnegie Mellon University, UNITED STATES

## Introduction

Marginalized groups, including Black, Indigenous, and people of color (BIPOC), members of the lesbian, gay, bisexual, transgender, queer or questioning, and two-spirit (LGBTQ2S+) community, people who are deaf, people who have disabilities, people from low-income backgrounds, and women continue to be severely underrepresented in science. Inequality with respect to access, retention, and success in science arise from overlapping systemic barriers that disadvantage historically underrepresented groups [1] while benefitting the dominant group of white, cisgender, and straight men in science. Barriers for historically underrepresented groups include ongoing racism [2], sexism [3], discrimination based on sexual preference or gender identity [4], ableism [5], socioeconomic disparity [6], and exclusionary learning environments [5,7]. These issues can compound to exacerbate the difficulty historically underrepresented groups face in achieving scientific success. For example, women of color are subjected to increased gender- and race-based harassment in astronomy and planetary science compared to white women, white men, and men of color [8–10], and LGBTQ2S+ STEM students are negatively impacted by sexist educational environments due to the interconnectivity of sexual orientation and gender stereotypes [11,12]. Given the persistent and overlapping systemic barriers faced by historically underrepresented groups, there is a clear and urgent need to find solutions that elevate all historically underrepresented groups in science [13].

Strategies that aim to dismantle barriers in science must ultimately come from all levels of the scientific community. As scientists and science educators, we believe that there are steps we can take to empower historically underrepresented students to succeed and persist in science. We derive these rules from our own lived experiences navigating our science educations as historically underrepresented and early-career scholars, as well as from the large body of science equity literature. The authors of this piece include educators who are BIPOC, queer individuals, women and nonbinary individuals, first-generation immigrants, and individuals with mental illness. Although all of the authors are currently studying as graduate and postdoctoral scholars at North American institutions, many were raised and educated around the world, including in the Bahamas, Bangladesh, Brazil, British Virgin Islands, and Lebanon. Our collective teaching experience includes creating and delivering undergraduate and graduate courses at universities, supervising undergraduate research students, teaching at the elementary and high school level, and creating or working for private and nonprofit outreach programs, including those that specifically target historically underrepresented students [14].

Here, we present 10 pedagogical strategies that can be employed by teaching scientists to elevate historically underrepresented students. This piece is directed at those who teach at universities and community colleges, museums, and other science-based organizations, as well as scientists who mentor students through research (e.g., as supervisors) and science outreach programs. Broadly, these rules can apply to postsecondary students attending universities and community colleges (e.g., through teaching courses), graduate students (e.g., through supervisory roles), as well as primary and secondary students (e.g., through science outreach). Ultimately, as scientists and educators, leveraging our positions of power to reduce the barriers faced by underrepresented students will not only enhance access and equity for our students but will benefit science and broader society as a whole [15,16]. We note that historically underrepresented scholars have often led the charge and faced further marginalization for their commitment to equitable education [17], so it is especially critical that scientists and educators from dominant groups commit to elevating underrepresented students in science.

## Rule 1: Teach with empathy

During our time as educators, we will come across students from a variety of different backgrounds, lived experiences, and personal lives. Empathy, at least in this pedagogical sense, describes the ways in which we seek to understand and extend compassion toward these unique realities of our students [18]. Empathetic teaching approaches are especially beneficial for students from marginalized backgrounds, whose experiences may have included numerous social, cultural, and political barriers [19,20]. For example, mentors who seek to eliminate homophobic stigma by championing the use of affirming language and promoting safe spaces in their classrooms improve the experience of LGBTQ2S+ students [21]. Similarly, educators who develop learning environments based on cultural responsiveness and understanding (e.g., sensitivity toward particular cultural issues or barriers students may face) improve black students’ feelings of agency in the classroom [22]. We suggest empathy not only as a first rule, but one that should be practiced continuously, and integrated with other strategies discussed herein.

To begin, it is integral that we actively engage with our students on a closer, personal level. We must invite dialogues with our students that facilitate the sharing of their experiences and make an intentional effort to understand their perspectives [23]. In practice, we could accomplish this simply through opening the floor during the first few minutes of class to allow students to discuss ideas, events, or personal stories that are on their minds [22]. This form of culturally responsive teaching may prove particularly useful during urgent local or global events that directly affect students (e.g., Black Lives Matter movements or the COVID-19 pandemic). In addition to addressing our students empathetically in groups, we should also be willing to resign traditional methods in favor of individual approaches that improve individual outcomes. For instance, headphones may be discouraged in the classroom as a general rule, but a student with attention deficit hyperactivity disorder (ADHD) may significantly benefit from playing white noise in the background [24]. Rather than telling the student what they should do (“remove the headphones”), we should seek to understand the individual student’s needs (“headphones help me focus”). It is also important to note that demonstrating empathy in the classroom is an ongoing process that also involves cycles of feedback from students [25], which we should invite continuously and informally throughout the term [26]. As we continue to expand our knowledge of marginalized students’ perspectives, we must be willing to renegotiate our own approaches and behaviors to prioritize kindness. Ultimately, this may mean that we, as educators, must challenge existing pedagogical frameworks that systematize the learning process to the detriment of equity.

## Rule 2: Implement student-centered learning

Student-centered learning (SCL) is a progressive teaching style that shifts the focus of instruction from teacher to student; centering what and how students want to learn [27]. Under SCL, students become active participants in their learning and are given a choice in what they will learn, their learning pace and style, and how they will assess their own learning [28]. SCL has been shown to benefit students through enhanced student autonomy, engagement, confidence, critical thinking, reflecting thinking, problem solving, and a more meaningful learning experience and has specifically been shown to increase retention and success of historically underrepresented students [29,30].

There are several ways that we can employ SCL to benefit historically underrepresented students. In particular, we can impart historically underrepresented students with freedom to choose what and who to study, allowing them to navigate science based on their varying cultural and philosophical foundations, which are often neglected in scientific pedagogy [7,17]. For example, under a traditional teacher-centered learning approach, we might ask students to submit an assignment on an influential scientist such as Charles Darwin, whose identity aligns with the experiences of majority groups. Whereas, under SCL, we would give each student the autonomy to choose scientists whose identities may better align with their own. As educators, we can further suggest diverse scholars to aid students in accessing important marginalized voices. In this scenario, an Indigenous student may instead choose Robin Wall Kimmerer, an Indigenous botanist who blends Western and traditional ecological knowledge to understand plants and the environment. More broadly, having autonomy over what and who to study can lead to increased creativity and learning outcomes for all students [29,30].

SCL also relies on active learning strategies, whereby students are experientially involved in their learning process through in-class discussion activities and low-risk assessments [31]. Although active learning pedagogy increases learning and performance of all students [31], it disproportionately benefits historically underrepresented students in particular [32–34]. Active learning strategies are linked to increased GPAs of historically underrepresented students in science, which, in turn, lead to higher retention rates [35]. This reduction in the achievement gap may occur because frequent low-risk assessments (in lieu of a few high-risk assessments) can prevent underrepresented students from entering the “danger zone” (i.e., a low course grade), which can otherwise lead to dropping out of science courses and programs [35,36]. Active learning strategies are also more conducive to high-quality, inclusive, and individualized learning environments that can benefit underrepresented students, who may otherwise struggle under more traditional passive learning settings [35]. We note that if student pairs or groups are created to facilitate active learning, they should be created conscientiously to prevent microaggressions (e.g., through letting students suggest peers to work with or balancing groups by historically underrepresented composition [37]).

Our implementation of SCL can further benefit historically underrepresented students by allowing them to have a voice in how they are assessed [38], which, in turn, leads to more flexible assessment styles that accommodate varied student needs. For example, students suffering from social anxiety may prefer a written assignment over an oral presentation, whereas a student with a writing disability may prefer an oral presentation over a written assignment. Tests and exams can also be made more accessible to English language learners through varied assessment options. For example, essay questions on exams can give students the option to answer in paragraph format, or by concept mapping with terms and illustrations [37]. Educators can also implement ungrading, a recent assessment style where instructors provide ungraded and extensive feedback to students and jointly come to a consensus with each student on what their final grade should be, with some ungrading proponents advocating for students being the sole decider of grades [39,40]. Ultimately, increasing students’ voices on assessments can reduce barriers around academic achievement and can encourage all students to be more creative, reflective, and empowered about their learning.

Collectively, SCL allows us to accommodate the varying needs of students, which is particularly beneficial for historically underrepresented groups. Given its many benefits, we encourage scientists to implement SCL into their teaching style, whether it be through a gradual integration [26] or through full-scale adoption of this approach [41]. SCL strategies can also be implemented in larger classrooms, for example, by offering student-centered assignment topics and assessment styles, and using pair and group work to encourage cooperative learning [42,43].

## Rule 3: Facilitate student empowerment

Discrimination and oppression that continue to permeate learning spaces can have numerous negative consequences for students from historically underrepresented backgrounds. For example, students may face stereotype threat, the development of anxiety and concern that they may fall into existing negative stereotypes about their group [44]. Students from historically underrepresented backgrounds may also feel as if they are excluded from or do not belong in academic spaces [45]. These psychosocial factors directly linked to students’ marginalization in society can ultimately have serious and negative impacts on both their well-being and scholastic achievement [44,46]. It is thus imperative that, in our role as educators, we strive to equip students with tools for breaking down psychosocial barriers by encouraging student empowerment.

One simple but effective strategy that we can implement in our classrooms is “values affirmation,” which affirms the self-worth, self-integrity, and, ultimately, emotional fortitude of students [47]. Values affirmation can be a brief classroom exercise that invites students to write a short statement about why certain values (e.g., athleticism, community, independence) are important to them (a published format for this exercise can be found in [48]). This simple intervention can empower students by lessening stereotype threat, leading to increased school performance and student sense of belonging [48,49]. Notably, values affirmation can be useful in situations with large class sizes and need only take 15 minutes per exercise to see positive effects [48].

We can also encourage historically underrepresented students to develop empowerment through promoting their self-advocacy, which is a crucial lifelong skill that students can use to overcome systemic challenges throughout and beyond their education [50]. To start, we should begin introductions to our educational programs by clearly stating that students have a right to assert their needs and receive support, whether to us personally or to the institution, and by emphasizing the institutional supports that are available. Implementing this first step may be especially useful for historically underrepresented students, who can face systemic barriers that discourage them from self-advocating. We should facilitate student self-advocacy by providing venues through which students can seek support, such as an anonymous online question box [50], or through making space for one-on-one discussions (e.g., office hours). When students self-advocate or provide feedback to us, we must make an effort to respond positively and with humility, making appropriate changes to the language or approaches we use in learning spaces. Positively incentivizing students to self-advocate could involve commending the student for speaking up and making a deliberate effort to empathetically listen to the student’s concern. Continuing to follow through with the student is also critical. For example, if a student expresses concern over their ability to attend class in person, an educator could make materials available online, rather than punishing the student for attendance [51].

Importantly, we can facilitate student empowerment through reducing student–teacher power imbalances. The student–teacher power dynamic is already steeply imbalanced, especially in cases where the instructor may be from a majority background, leading to barriers to student empowerment. Reducing this power imbalance can encourage students to take active roles in their own learning. For example, we can explicitly state that we welcome students to question the material we present and follow up by engaging in informal and positive discussions if students choose to do so [51,52]. We can also employ SCL (Rule 2) to actively incorporate student ideas into lesson plans, effectively inviting them to co-create their own material [26,53]. Co-creation of learning could take the form of asking students to choose curriculum-relevant lecture topics or examples, especially those with more relevance to their own lived experiences. In reducing power imbalances, we can encourage our students’ belief that they are meaningful contributors in their own educations and thus empowered within their learning spaces [54].

## Rule 4: Diversify scientific perspectives

Another way to engage effectively with historically underrepresented students is to diversify the perspectives through which they learn. Science is typically taught through the lens of certain paradigms, which are, for example, largely Western [55] and male biased [56]. These majority paradigms may not ultimately reflect the diverse perspectives of students themselves, causing a disconnect between learning material and their own experiences. Educators can seek to broaden the scientific perspectives discussed in their classrooms by hosting and working with diverse leaders and by adopting culturally mediated teaching styles.

Historically underrepresented students often lack a true sense of belonging at their institutions; the general scarcity of people in leadership positions who look like them, stereotype threat, and imposter syndrome make it difficult to feel welcomed [45,57]. Educators can help reduce these negative pressures by hosting diverse guest speakers and hiring diverse teaching assistants and workshop leaders. In addition to delivering educational material from unique perspectives, these guests can also share personal stories about their backgrounds, career trajectories, and some of the barriers and challenges they face. When students find a common ground with their teachers and mentors (e.g., similar demographic characteristics), they feel more cared for and become more motivated to learn [58]. Small changes like these ultimately provide an environment in which historically underrepresented students see tangible evidence of what success can look like for them and become more inspired to undertake leadership roles in the future. Ultimately, academic institutions should ensure that underrepresented scholars are well represented across leadership positions. If, as educators, we rely on equitable representation through other means (e.g., guest lectures), we should be conscientious that underrepresented scholars are often inundated with such request and ensure that their time is valued and rewarded if they decide to take on these roles.

Diverse instruction also widens the perspectives through which students with different backgrounds and unique learning styles can acquire new knowledge. Educators should focus on using culturally mediated analogies and relatable examples to explain scientific concepts to their students and mentees as a culturally sensitive form of differentiated instruction [59]. For example, skiing might be a great example to explain low friction for some students, but this same example would not resonate with students who have never had the opportunity to ski, an activity that is both geographically and cost prohibitive. Here, taking multiple approaches to explain a concept would greatly improve knowledge transfer. Additionally, having multiple scientific perspectives reduces biases [60]. For example, people in inland North America might generally feel that they are not directly or strongly impacted by climate change, and, as a result, subconsciously underestimate climate change effects on coastal communities and other heavily impacted groups. By being exposed to varied experiences and scientific perspectives through diverse speakers, books, documentaries, and other mediums, students and educators alike can develop a better understanding of climate change impacts and a deeper appreciation of how science relates to all people and cultures.

## Rule 5: Reduce financial barriers

Financial barriers continue to negatively influence the retention and success of low-income students, who often come from other historically underrepresented groups, such as BIPOC communities [6]. Low-income students often lack parental and family engagement and have inadequate geographical and social environments, both of which perpetuate the achievement gap among marginalized communities. For example, financially constrained parents commit more time to their pressing financial needs, influencing their available time for supporting their children’s educational routines and school engagement [61]. In the geographical and social context, food deserts are common barriers that affect individuals of lower socioeconomic statuses, which negatively influence educational outcomes [62]. Further, students who live in at-risk communities or who are homeless often lack many important resources such as access to community centers, safe playgrounds, libraries, and enrichment programs, which negatively affect the development of low-income individuals in social, emotional, and cognitive aspects [62]. Collectively, these issues are perpetuated from the individual to their community and, later, to society, thus creating intergenerational problems.

As educators, we should replace or adapt our practices to be responsive to the needs of low-income students. First, we must undergo deep self-reflection and shift our conception of financially constrained students from being deficit based to asset based, an approach that demonstrates appreciation for the work and sympathy toward the struggles of low-income students [63]. For example, a student’s goal might be to pass a class because they are working a full-time/part-time job to make ends meet. Instead of focusing on how the student’s performance in the class would be better if they were not working, acknowledge that student’s ability to balance school and work. In turn, this approach should reduce our biases toward these students and give us ideas about how to meet their needs [63,64]. For example, given that low-income students may need to work on top of their studies [65], educators can ensure that classwork and assignments can be completed in a reasonable amount of time and that additional time commitments (e.g., office hours) are not mandatory for academic success in a course.

Importantly, we must be aware of and accept that low-income students rely heavily on educational institutions to develop skills that support their academic achievement and aim to create lesson plans that reduce or preclude financial barriers. As a baseline, we should not assume that students have access to resources like transportation and costly materials and consider the associated costs when creating lesson plans. More generally, to increase financial accessibility, we should prioritize open-source software and texts and inexpensive materials in lieu of high-cost lessons when possible. For example, educators can support an open science concept, such as Wikipedia editing, or a group domain, as a way to have students work together to contribute to and reframe knowledge. We can also encourage our students to work together in ways that share limited materials, thus reducing the financial burden on individuals (e.g., organize a carpool program to a rural field site). If specific materials are needed, we should make them freely available to students in a discreet way, use resources as a class (e.g., visit the computer lab during class or tutorial time), or petition our institution to provide the required materials to make our classes more accessible. More broadly, we can advertise available funding opportunities to our students, particularly scholarships with diversity components (i.e., targeted funding for low-income and other historically underrepresented groups). These opportunities may provide critical financial aid to low-income students as they continue their educational journey [17].

## Rule 6: Advocate for and create accessibility in learning environments

People with disabilities continue to face significant barriers that negatively impact their educational representation and success [66,67]. Although improvements have been made over the past decades [68–70], students with disabilities (SwD) still face cultural/societal (including stigmatization, prejudice, stereotyping) and physical (e.g., limited access to buildings or spaces due to architectural impediments) barriers while trying to successfully achieve educational goals. Exacerbating these difficulties are bureaucratic complexities surrounding accessibility. Institutions acknowledge learning accessibility in different ways, with different levels of commitment in prioritizing accessibility, as well as available resources to invest in improvements [68,71]. Even though most factors to ensure accessibility occur at an institutional level (e.g., structural modifications, trained support personnel, assistive technology, scholarships), as educators, we also play a pivotal role in creating accessible learning environments and academic experience for SwD, even in institutions where resources are scarce and/or accessibility is not a priority.

We can start by facilitating SwD to engage in self-determination [72], by describing available resources offered by the institution (e.g., faculty/lab tutors, interpreters, focus groups, academic assistance), and by assisting students with navigating these often-complex institutional systems [73,74]. When resources are not available, we should aim to meet with institutional representatives to reinforce the need for the adoption of universal design principles [75], including structural renovations, auxiliary tools, and/or disability support services (e.g., professional development, qualified interpreters, elevators, adapted computer terminals). In class, we should always consider class location, requesting accessible classrooms, laboratories, and field areas. Throughout this process, it is key that we discuss accessibility needs and preferences with our students [73]. One concrete idea is to include questions about preferences, access, and accommodations in a survey given to students prior to the beginning of the term. It is also essential to make reasonable adjustments when necessary, which may require flexibility in our teaching approach and assessment [74,76]. Ultimately, educators should have a working knowledge of legal requirements for SwD at both institutional and federal levels [77]. When well informed, we can better accommodate and advocate for SwD, as well as develop best practices that go beyond legal requirements in regions where those are low or absent. Importantly, we should remain compassionate and show a positive attitude toward SwD, as instructor attitudes toward SwD can play an essential role in their academic success [74].

## Rule 7: Connect students with resources and opportunities

As educators, we can assist our students with navigating educational systems by connecting them with resources and opportunities that remove barriers or elevate their STEM education and careers. Students from historically underrepresented backgrounds may significantly benefit from these resources, but due to factors such as unfamiliarity with complex institutional structures, these resources may be difficult to access. Further, spending time looking for resources adds additional work to the plates of historically underrepresented students [78,79].

Resources could include financial opportunities, such as paid research positions, or funding and scholarship opportunities aimed at historically underrepresented students [80,81]. We note that educators should normalize and advocate for students to take on paid positions over volunteer work, whenever possible [17]. Educators should also encourage historically undergraduate students to pursue available research opportunities available at their institutions (e.g., honors thesis, summer research internships), facilitating their participation in science research, networking, and career development. Importantly, providing information on how to approach professors and apply for funded research opportunities may go a long way for students who are not familiar with the process. Historically underrepresented students may also significantly benefit from learning resources, such as writing centers, writing workshops, and libraries; we should aim to connect students with these tools as early as possible. Other co-curricular opportunities frequently offered by institutions should be widely publicized to students, including career panels or lectures, networking opportunities with visiting or institutional researchers, and seminars. Lastly, equity resources are particularly important to historically underrepresented students, including diversity-focused events and organizations. Equity resources can assist students in developing networks of peers and mentors who are also from historically underrepresented groups, increasing their feelings of belonging and success in science [45,82]. We encourage educators to keep track of these resources and advertise them in the class syllabus through class announcements or other means of communication (e.g., newsletters) to promote their uptake and, ultimately, the success of historically underrepresented students [6,82].

## Rule 8: Facilitate access to informal education and enrichment

A comprehensive understanding of the natural world cannot be achieved solely through education in formal environments like classrooms. It has been suggested, for example, that sources such as museums, national parks, and science centers have at least an equal role to play in science education compared to more structured curricula [83,84]. The effects of these venues in increasing long-term interest in science fields is more pronounced when engaging students early in their education [85], which, in turn, has positive effects for future success in these disciplines [86].

However, communities currently underrepresented in science often have significantly reduced access to critical informal education opportunities. Engagement in informal education, which is voluntary and regularly incurs financial cost, often hinges on parental familiarity, education level, and family socioeconomic status [87]; all 3 of these factors are likely to disproportionately disadvantage historically underrepresented students [88–91]. Therefore, any comprehensive plan to improve the representation of historically underrepresented groups in science disciplines should incorporate the huge potential of informal learning for inspiring motivation in students that are typically not encouraged in those fields.

To that end, we strongly encourage educators to incorporate informal learning environments into their lesson plans as fundamental course components. Science centers and museums are obvious choices that are applicable across all science subdisciplines. A holistic appreciation for the biological sciences in particular almost necessitates field experiences (including field-based experiments, nature walks, and diversity surveys), which have been shown to critical determinants of future success [92,93]. These experiences should be made available irrespective of the financial resources of the students and framed in such a way that students with less of a background in informal settings are not made to feel as though they are “behind” their peers (see rules 1 and 2). This is particularly true of students that may not have entered college or university directly out of high school, who often report different needs and expectations from postsecondary education [94,95]. A successful implementation of this strategy also requires that the informal learning experiences be designed and integrated into the course thoughtfully, providing informed guidance throughout rather than simply opening the door and letting students succeed or fail on their own. This integration of formal and informal education efforts has enormous potential to bridge the gap between the 2 models, combining the structure of the former with the self-motivating strength of the latter [96]. This is particularly true if long-term relationships can be established between lecturers in universities and science educators at informal education sites [97] and can be especially impactful for groups that have been historically excluded from these formative experiences. This further requires that educators be flexible and embrace new strategies and technologies to improve access to STEM education. This may include leveraging digital resources such as virtual museum tours (e.g., those available from the Smithsonian Institutional Natural Museum of Natural History and Aga Khan Museum), interactive science websites (e.g., Royal Ontario Museums’ Burgess Shale website) using freely accessible online STEM content (e.g., PBS eons), or by directly engaging science communicators online who can give guise presentations to students (e.g., Skype A Scientist). The often free (or relatively inexpensive) nature of these types of programs presents a significant breakdown of the socioeconomic barriers to science equity we described in rule 5.

## Rule 9: Integrate community

Scientific culture is often associated with individualistic work goals, a narrow and exclusive disciplinary culture, and a detachment from communities outside of science. As a result, scientific fields of study tend to attract and retain more students who value intrinsic motives (e.g., passion for problem solving and discovery, curiosity) and accept the individualistic and isolating aspects of the field [98]. Students from historically underrepresented minority groups also value intrinsic motives in their career interests but are much more likely than majority students to be influenced by highly altruistic cultural values, particularly contributing to one’s community [98,99]. The sense of community that is integral to many minority groups fosters cultural values and identities in students that center around promoting meaningful communal connections and “giving back” to their community [100,101]. Because minority groups are often disproportionately affected by social justice issues, they are likely more aware of the impacts and feel more urgency to act on these issues for the betterment of their community. Not all scientific fields afford or value communal work goals, and, as a result, students from historically underrepresented minority groups may be particularly at risk for losing interest in science careers [102,103]. Instead of changing historically underrepresented students to fit the majority, we need to change research training and science education to be more inclusive of their values [38].

Scientists and educators can integrate community into their teaching and mentoring activities by building links between culture and science that are particularly relevant to historically underrepresented students [104]. For example, climate change is an environmental issue that is widely addressed in scientific research and education for its global impacts. To make a meaningful connection between climate change and community, we can focus on how climate change disproportionately affects marginalized communities (e.g., pollution, food security, extreme weather events) and discuss why this makes their input and leadership in research even more valuable. Community-relevant topics can be integrated into the classroom by incorporating the diverse viewpoints and perspectives from historically underrepresented groups into traditional lecture topics (e.g., environmental justice in climate change, subsistence fisheries and food security in fisheries and conservation, Indigenous rights in resource management). As educators, we can develop class assessments that contribute to community science and/or outreach while meeting curriculum requirements (e.g., presentations are given to children’s groups or organizations rather than presented in class). In mentoring activities, we can implement SCL and support students wishing to pursue cultural or communal based research endeavors. Both in the classroom and through mentoring, utilizing an asset-based approach in which educators can draw upon the knowledge of their students (e.g., asking students to share the knowledge they have gained about science from their family or culture) helps integrate community experience and learning. We can also tailor our outreach to students from historically underrepresented minorities by facilitating a sense of community through long-term interactions and empowering students to engage in issues that directly affect their community. For example, community science platforms such as iNaturalist allow students to record their own observations and interact with members of the community to discuss and learn more about their findings, while also making direct and beneficial scientific impacts [105]. Educators in positions of power can investigate community and outreach opportunities (e.g., Soapbox Science, Science Literacy Week) and present them to their students to encourage involvement. As we strive to establish meaningful connections with communities, we must also be cognizant of the time-intensive nature of this process and ensure that we are not intentionally or unintentionally exploiting communities and organizations or engaging in nonreciprocal relationships. Employing community-oriented science not only attracts more students from diverse backgrounds and reduces a major barrier to successful assimilation into scientific culture, but it can lead to more fulfillment and drive in scientists to better both science and society.

## Rule 10: Commit to ongoing education and accountability

Scientific innovation requires constantly shifting our perspectives, reframing our ideas, and striving for excellence. This mindset should also be applied to our teaching strategy, where committing to ongoing equity education and accountability can support historically underrepresented scholars, who are disproportionately affected by economic, social, or systemic barriers [106].

As educators, we can commit to ongoing education both within our organizations and through self-learning. Equity, diversity, and inclusion (EDI) workshops and seminars are increasingly being instated by academic institutions with the objective of holding leaders accountable for advancing diversity [107,108]. These learning opportunities have been shown to cultivate more inclusive academic environments and contribute to the development of a more diverse workforce [109]. Educators are encouraged to seize these opportunities for development, solicit them from their institution if they are lacking, and engage with peers to normalize the exchange of ideas surrounding inclusivity. In sharing, we can develop solutions that highlight unique identities, backgrounds, and experiences. This formation is not limited to the workplace. At a personal level, educators should be dedicated to ongoing learning outside of the classroom. This could include an increased awareness of our own biases and assumptions about others [107,110], as well as recognizing challenges faced by different races and ethnicities, genders, identities and orientations, socioeconomic statuses, and disabilities to tailor teaching approaches appropriately (rules 1, 2, and 3). Awareness could also involve keeping up with current global events that may impact student’s well-being. This includes consulting reliable news sources covering a range of global issues (e.g., politics, economics, ethics, natural disasters, and crime), critically thinking about how these occurrences may affect our students and being prepared to engage in supportive discussions surrounding these events. Resources tailored to EDI are available in many forms (books, seminars, videos, infographics, scholarly material, etc.); material curated by academic institutions may be a good place to start (e.g., see [17,110]).

Creating a culture of accountability in the workplace applies not only to ourselves—it extends to others. For example, historically underrepresented students are often the targets of microaggressions [17], which are prejudicial comments or actions, often delivered in an offhand manner [111]. Instead of “calling-out” and shaming the perpetrator of microaggressions, it can be reframed as an opportunity to learn and discuss the behavior, why it occurred, and the effect that it may have by instead “calling-in.” This term was coined by Loretta Ross, and unlike calling-out, calling-in is “speaking up without tearing down” [112]. It is conducted with respect and invites conversation and compassion. Normalizing these often-difficult conversations can lead to increased collective understanding and inclusiveness. Unfortunately, there may also be situations in which a prejudicial behavior of a student or even faculty member is serious or dangerous. In these circumstances, we also need to ensure that we are familiar with our institution’s reporting systems in order to guide and support historically underrepresented students in navigating the situation. Committing to ongoing education applies to our responsibility not only as scientists but also as educators. In doing so, we uplift important and diverse perspectives that will continue to pave the path of scientific discovery.

## Concluding thoughts

Historically underrepresented students continue to face a myriad of barriers that limit their success and retention in science. As educators, we must do our part to provide a more inclusive and empowering environment where diverse students can thrive. Increasing representation and equity within science is necessary for ethical and moral reasonings and will lead to the betterment of both scientific discoveries and science culture.
